# Supplementary material for: Lmx1a-Dependent Activation of miR-204/211 Controls the Timing of Nurr1-Mediated Dopaminergic Differentiation
Source: Int J Mol Sci. 2022 Jun 23;23(13):6961. doi: 10.3390/ijms23136961 (PMC9266978; doi:10.3390/ijms23136961)
Supplement: Supplementary file 1 [file ijms-23-06961-s001.zip › ijms-1759030-supplementary.pdf]

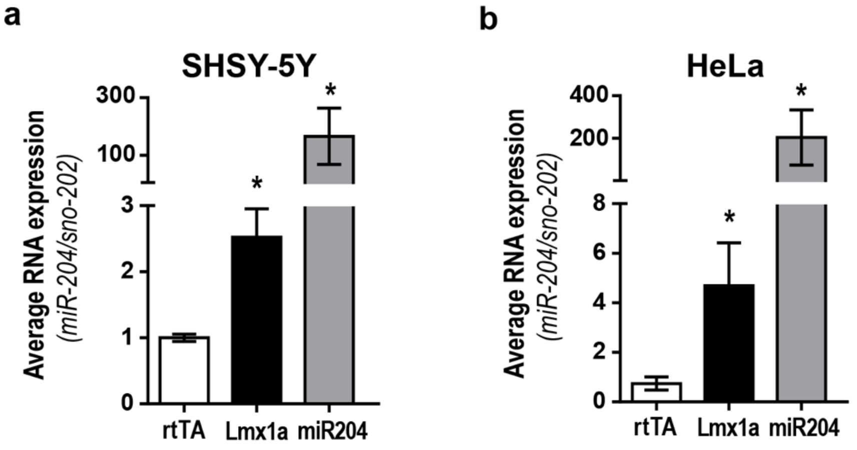

Figure S1: Lmx1a promotes miR-204/211 expression in human cell lines.
TaqMan assay for miR-204 on SHSY5Y (a) and HeLa (b) human cell lines. miR-204 values are normalized on the reference sno-202 Data represent the mean ± SD of 2-DDCt values from three independent experiments. *p < 005 with respect to Ctrl (one-way ANOVA + Tukey post hoc-test).

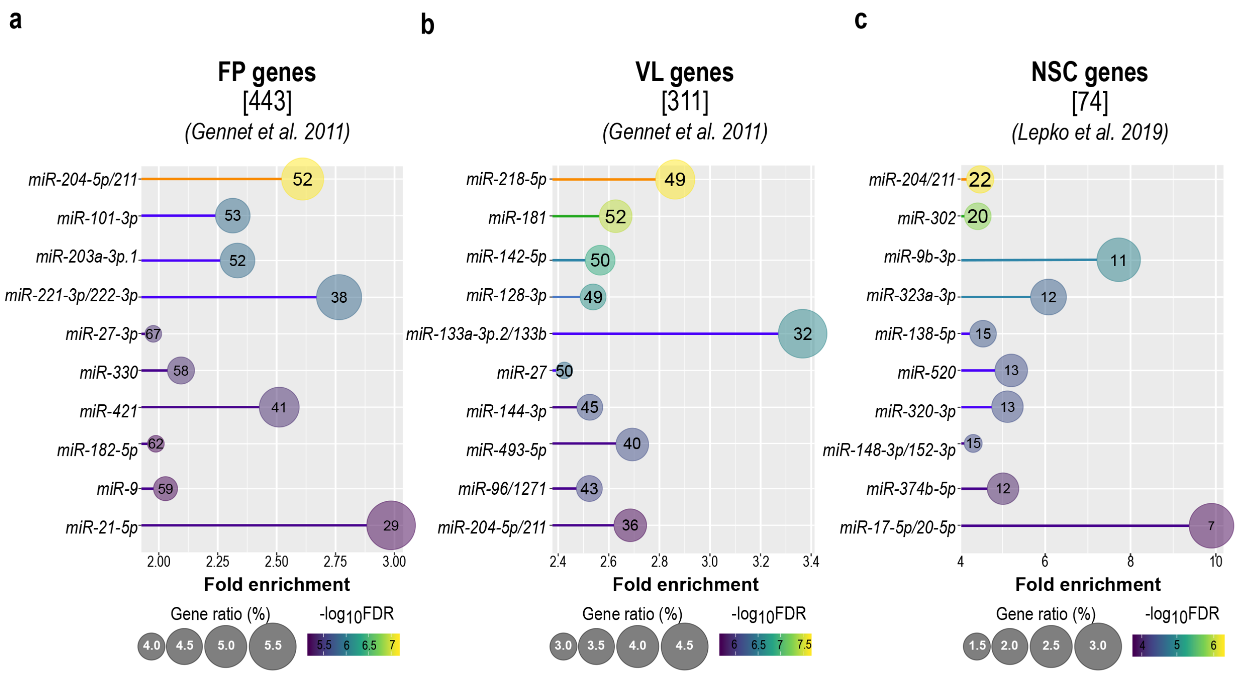

Figure S2: Genes involved in mid-brain development and highly predicted targets of miR-204/211. a-c) miRNAs' binding sites enrichment analysis on floor plate (FP) (a), lateral ventral (LV) (b) and neural stem cell (NSC) (c) specific genes (Gennet et al., 2011; Lepko et al., 2019). Enrichment analysis was performed with TargetScan prediction score using ShinyGO v0741 web-tool (http://bioinformatics.sdstate.edu/go/) (Ge et al., 2020). Lollipop-plots representing the fold enrichment for miRNA-target genes on the x-axis, while the bubbles-size represent the percentage of predicted genes on the total target for each miRNA. Absolute numbers of predicted genes are included in the bubbles. Enriched miRNAs are ordered for the -log10FDR values, represented as color gradient.

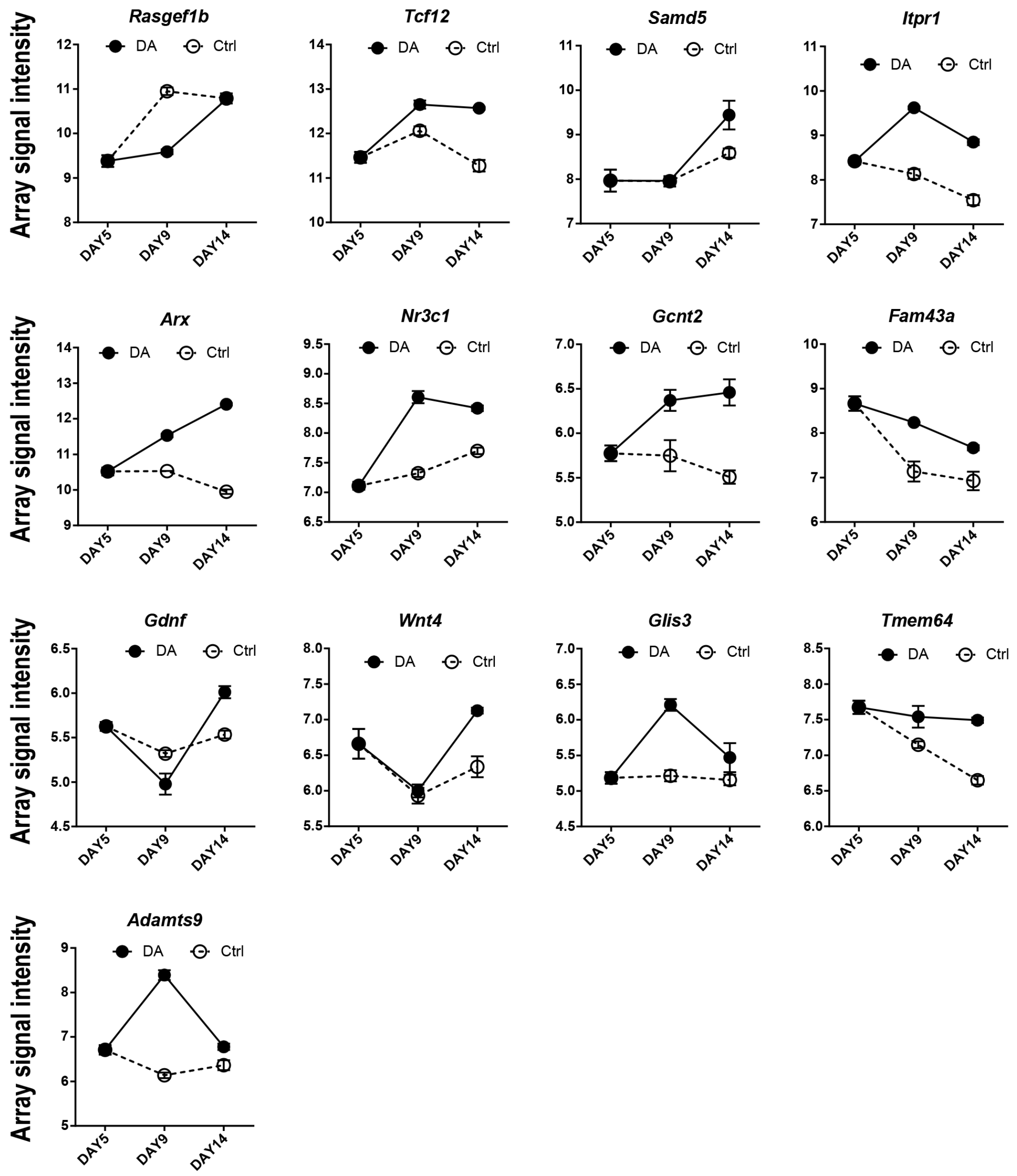

Figure S3: Expression pattern of miR-204/211 target FP genes during in vitro dopaminergic differentiation of mouse epiSCs.

Array values for selected target genes among the floor plate signature during in vitro DA differentiation of epiSCs (Gennet et al., 2011; De Gregorio et al., 2018). Data represent the mean ± SD of array values for triplicate samples.)

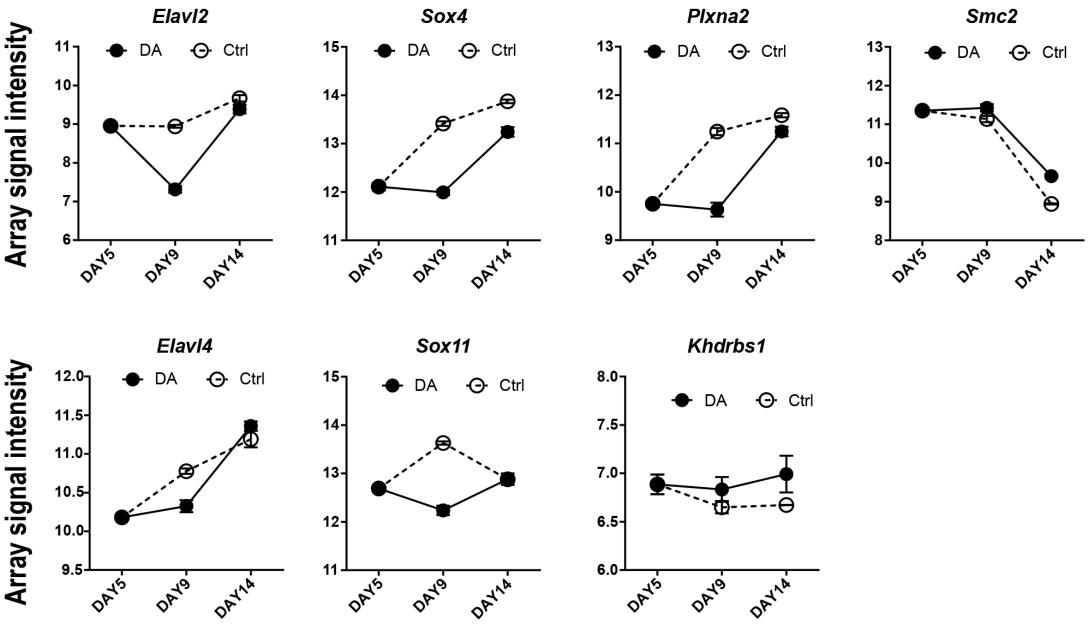

Figure S4: Expression pattern of miR-204/211 target NSC genes during in vitro dopaminergic differenti-ation of mouse epiSCs.

Array values for selected target genes among the neural stem cells signature during in vitro DA differentiation of epiSCs (Lepko et al., 2019; De Gregorio et al., 2018). Data represent the mean ± SD of array values for triplicate samples)
